# The expression of cytokeratin 19 in lymph nodes was a poor prognostic factor for hepatocellular carcinoma after hepatic resection

**DOI:** 10.1186/1477-7819-11-136

**Published:** 2013-06-12

**Authors:** Chao-Wei Lee, Wen-Ling Kuo, Ming-Chin Yu, Tse-Ching Chen, Chi-Neu Tsai, Wei-Chen Lee, Miin-Fu Chen

**Affiliations:** 1 The Department of Surgery, Chang Gung Memorial Hospital, No 5 Fusing Street, Taoyuan County, Gueishan 333, Taiwan; 2 The Department of Pathology, Chang Gung Memorial Hospital, No 5 Fusing Street, Taoyuan County, Gueishan 333, Taiwan; 3 Chang Gung University, 259 Wen-Hwa 1st Road, Taoyuan, Kuei-Shan 333, Taiwan; 4 Graduate Institute of Clinical Medical Sciences, Chang Gung University, 259 Wen-Hwa 1st Road, Taoyuan, Kuei-Shan 333, Taiwan

**Keywords:** Hepatocellular carcinoma, Lymph node metastasis, CK19

## Abstract

**Background:**

The expression of CK19 in primary hepatocellular carcinoma (HCC) is associated with a poor outcome. However, few studies have investigated the expression profile of CK19 in regional lymph nodes (LNs) of HCC after hepatic resection. The purpose of this study was to evaluate the expression of CK19 in primary liver tumor and regional LNs of HCC with and without lymph node metastasis (LNM).

**Methods:**

The expression of CK19 in patients with (n = 16) and without LNM (n = 26) was examined using immunohistochemical staining. Both the primary tumor and LN specimen were studied for their CK19 expression. Clinico-pathological variables and prognostic significance were analyzed.

**Results:**

Immunopositivity of CK19 in primary liver tumor was significantly correlated with LNM (*P* = 0.005) and tumor non-encapsulation (*P* <0.005). Univariate analysis showed that CK19 expression in primary liver tumor, CK19 expression in regional LN, vascular invasion, daughter nodules, positive resection margin and American Joint Committee on Cancer (AJCC) tumor stage significantly decreased overall survival. Multivariate analysis demonstrated that daughter nodules (*P* = 0.001) and CK19 expression in regional LN (*P* = 0.002) were independent prognostic factors for overall survival.

**Conclusions:**

This study showed that CK19 expression in regional LN of HCC was associated with LNM and an extremely poor outcome after operation. It is of clinical significance to identify these patients at risk for more aggressive HCC, and multi-modality treatment could be helpful to improve their dismal outcome.

## Background

Hepatocellular carcinoma (HCC) is the most common primary malignancy of the liver with an estimated annual death incidence of approximately 600,000 worldwide. In Taiwan, it is the second most common cause of cancer death and causes more than 7,500 deaths each year [1]. Surgical resection remains the most effective therapy in selected patients, but approximately 75% of patients with HCC have advanced unresectable diseases upon presentation. Compared with other malignancies such as lung cancer, esophageal cancer, renal cancer, gastric cancer, and intra-hepatic cholangiocarcinoma, the incidence of lymph node metastasis (LNM) in primary HCC is very low, and the prognosis is poor when LNM occurs [2-11]. Nevertheless, despite this poor outcome, previous study has documented that the 5-year overall survival rate of HCC with LNM after hepatectomy and lymph node dissection was 13.6%, and 5 out of 22 patients had survival of more than two years [12]. Due to LNM’s dismal role in prognosis, it is imperative to identify those patients at risk for LNM for more aggressive treatment and detailed examination.

Cytokeratin19 (CK19) is a biliary epithelial cell marker and is generally expressed in intrahepatic cholangiocarcinoma (ICC) cells [13]. Studies have shown that the expression of CK19 in primary HCC is associated with poorer outcome [14]. There have also been studies demonstrating that CK19 expression in primary HCC is a significant risk factor for developing LNM [15-17]. However, few studies have investigated the expression profile of CK19 in regional lymph nodes (LN) of HCC after hepatic resection. HCC with CK19 expression, speculatively, may be a special subtype of HCC with distinct clinical behavior from HCC without CK19 expression. Therefore, the purpose of this study was to examine the expression of CK19 in primary liver tumor and regional LN of HCC with and without lymph node metastasis. The correlation between CK19 expression in LN and LNM was determined. Clinico-pathological variables were also investigated to explore the impact of CK19 expression on survival outcome. Finally, the significance of HCC categorization according to CK19 expression was elaborated.

## Methods

### Patients

For the years from 1982 to 2010, records of patients with histological proven primary HCC from the Cancer Registry of the Cancer Center, Chang Gung Memorial Hospital, Linkou, Taiwan, were retrospectively reviewed. Only patients who underwent curative hepatectomy by the same surgical team were eligible for this study, and their clinico-pathological data were retrieved from the prospectively collected database. The following variables were included in the analyses: age, gender, cigarette smoking, alcohol consumption, hepatitis B virus (HBV) infection, anti-hepatitis C virus antibody (anti-HCV) level, alkaline phosphotase level, bilirubin level, preoperative alpha-fetoprotein level, Child-Pugh classification, tumor size, tumor-LNM status, tumor encapsulation, histological grade, fatty liver, resection margin, and mortality. The study endpoint was 30 June 2010, and tumor staging was based on the 6th edition of AJCC TNM staging system for HCC. This study was approved by the Institutional Review Boards (IRB 99-1127B) of Chang Gung Memorial Hospital (CGMH).

The indications for surgery included a lack of cancerous thrombi in the main trunk of the portal vein, no distant metastasis to other organs, absence of ascites or jaundice, and a technically operable main tumor in the preoperative evaluation [18]. Suspected LNM restricted to the hepatoduodenal ligament, detected by a preoperative image study, was defined as HCC operable by experienced surgeons, and hepatectomy combined with lymph node dissection was performed. For resectable tumors, the hepatic hilum and hepatoduodenal ligament were carefully examined and palpated to detect any enlarged lymph nodes by the chief surgeons. Lymphadenectomy was performed if there were enlarged lymph nodes. Forty-two patients who received lymph node dissection or sampling were selected from our database for this study. Of them, sixteen patients (38.1%) had pathologically proven LNM and 26 (61.9%) did not have LNM. Absence of LNM was confirmed by the following three criteria: (1) negative reports of preoperative computed tomography scans, interpreted by experienced radiologists; (2) no intra-operative detectable enlarged lymph nodes, proven by experienced hepatobiliary surgeons; and (3) negative postoperative pathological report of LNM in the resected specimen, examined by pathologists who were experts in hepatology.

### Immunohistochemistry

Formalin-fixed and paraffin-embedded resection specimens were sectioned to 4µm in thickness and deparaffinized, rehydrated, and processed for antigen retrieval. The slides were further incubated with diluted (1:100) monoclonal antibody to CK19 (Abcam, San Francisco, CA, USA) at room temperature for 1 hour. After incubation, the slides were washed three times in phosphate-buffered saline, incubated with a horse reddish peroxidase conjugated antibody (ZYMED, San Francisco, CA, USA) at room temperature for 10 minutes, and then developed by treatment with 3,3-diaminobenzidine (Dako North America, Inc. Carpinteria, CA, USA) at room temperature for another 10 minutes. Independent experienced pathologists without knowledge of patient characteristics and outcome determined the results of immunohistochemical staining under microscopy. Both the primary tumor and LN specimen were subjected to study, and positive CK19 expression was defined as ≥5% of tumor/LN cells stained positive for CK19 [15].

### Statistical analysis

The statistical analysis was performed with IBM SPSS Statistics 21 (IBM Corporation, Software Group, Somers, NY, USA). Fisher’s exact test and Pearson’s χ2 test were used to analyze categorical data. Student’s *t* test was used to analyze quantitative variables. Overall survival (OS) was defined by the time elapsing from the date of diagnosis to either the date of death or the date of the last contact. Cases with surgical mortality, defined as death within one month of surgery, were excluded from the survival analyses. Kaplan-Meier analysis was used to determine the OS. The log-rank test and Cox regression multivariate analysis were applied to determine prognostic significance of clinic pathological variables. Statistical significance was defined as *P* <0.05.

## Results

There were 34 (80.95%) males and 8 (19.05%) females. Twenty-eight (66.67%) patients were HBV carriers and 9 (21.42%) patients had chronic HCV infection. As for the severity of liver cirrhosis, 41 (97.62%) patients were Child-Pugh classification A whereas only one patient (2.38%) had Child-Pugh classification B liver cirrhosis. In terms of tumor T stage, there were 12 (28.57%) T1, 6 (14.29%) T2, 18 (42.86%) T3, and 6 (14.29%) T4 patients.

Table 1 summarizes the relationship between clinicopathological variables and CK19 expression in HCC. CK19 expression was positive in nine (21.43%) primary liver tumor specimens and five (11.90%) regional LNs; immunopositivity of CK19 in primary liver tumor was significantly correlated with CK19 expression in LN (Spearman correlation coefficient 0.704, *P* <0.001). Statistical analysis showed that LNM (Spearman correlation coefficient 0.427, *P* = 0.005) and tumor non-encapsulation (Spearman correlation coefficient 0.382, *P* = 0.013) were significantly related to CK19 expression in primary liver tumor. On the other hand, patient age, gender, cigarette smoking, alcohol consumption, hepatitis viral status, preoperative total bilirubin, preoperative AFP, preoperative alkaline phosphatase (ALK-P), Child-Pugh classification, tumor size, T stage, tumor rupture, vascular invasion, daughter nodules, and histologic grade were not related to CK19 expression in primary liver tumor.

**Table 1:** The relationship between clinicopathological variables and CK19 expression in hepatocellular carcinoma

	**Hepatocellular carcinoma, n = 42**					
	** CK19 liver **^**a**^			** CK19 lymph node **^**b**^		
	**Negative**	**Positive (%)**	***P *value**	**Negative**	**Positive (%)**	***P *value**
**Age (yr)**						
≦**60**	24	5 (17.2)	0.275	26	3 (10.3)	0.497
>**60**	9	4 (30.8)		11	2 (15.4)	
**Gender**						
**Male**	26	8 (23.5)	0.443	30	4 (11.8)	0.673
**Female**	7	1 (12.5)		7	1 (12.5)	
**Hepatitis B virus**						
**Positive**	24	4 (14.3)	0.117	26	2 (7.1)	0.197
**Negative**	9	5 (35.7)		11	3 (21.4)	
**Hepatitis C virus**						
**Positive**	7	2 (22.2)	0.633	8	1 (11.1)	0.712
**Negative**	26	7 (21.2)		29	4 (12.1)	
**Total bilirubin(mg/dl)**^**c**^	1.348 ± 2.373	0.844 ± 0.335	0.533	1.303 ± 2.236	0.760 ± 0.270	0.595
**Alkaline phosphotase (U/L)**^**c**^	111.4 ± 70.447	93.86 ± 30.905	0.527	111.63 ± 68.54	85.40 ± 26.444	0.408
**Preoperative α-fetoprotein (ng/ml)**^**c**^	8300 ± 39207	669.42 ± 1258	0.589	7594 ± 37510	1033 ± 1525	0.701
**Child-Pugh classification**						
**A**	32	9 (21.9)	0.786	36	5 (12.2)	0.881
**B**	1	0 (0)		1	0 (0)	
**Size (cm)**	9.027 ± 5.2192	6.156 ± 3.0121	0.124	8.519 ± 5.1876	7.620 ± 2.7472	0.708
**T stage**						
**T1/T2**	13	5 (27.8)	0.524	16	2 (11.1)	0.688
**T3/T4**	20	4 (16.7)		21	3 (12.5)	
**N stage**						
**N0**	24	2 (7.7)	0.005	26	0 (0)	0.002
**N1**	9	7 (43.8)		11	5 (31.3)	
**Encapsulation**						
**Yes**	19	1 (5)	0.013	19	1 (5)	0.203
**No**	14	8 (36.4)		18	4 (18.2)	
**Tumor rupture**						
**Yes**	4	0 (0)	0.366	4	0 (0)	0.590
**No**	29	9 (23.4)		33	5 (13.2)	
**Vascular invasion**						
**Yes**	12	4 (25)	0.471	12	4 (25)	0.061
**No**	21	5 (19.2)		25	1 (3.8)	
**Daughter nodules**						
**Yes**	13	3 (18.75)	0.529	15	1 (6.25)	0.359
**No**	20	6 (23.07)		22	4 (15.4)	
**Resection margin**						
**Positive**	4	1 (20)	0.606	5	0 (0)	0.284
**Negative**	29	8 (21.6)		32	5 (13.5)	
**Edmonson and Steiner grade**						
**I/II**	15	4 (21.1)	0.180	18	1 (5.3)	0.341
**III/IV**	18	5 (21.7)		19	4 (17.4)	

^a^*Immunohistochemical staining of primary liver tumor specimen for CK19.*

^b^*Immunohistochemical staining of lymph nodes specimen for CK19.*

^c^*Data are presented as mean*±standard deviation.

As for lymph node examination, five (11.90%) patients had at least one regional LN positive for CK19 expression, and they all had CK19 expression in their primary liver tumor. All CK19 (+) LN were metastatic LN (N1 disease). CK19 expression in regional lymph node was therefore significantly correlated with LNM (Spearman correlation coefficient 0.469, *P* = 0.002). All of the other clinicopathological factors were not related to CK19 expression in regional LN.

**Figure 1:**
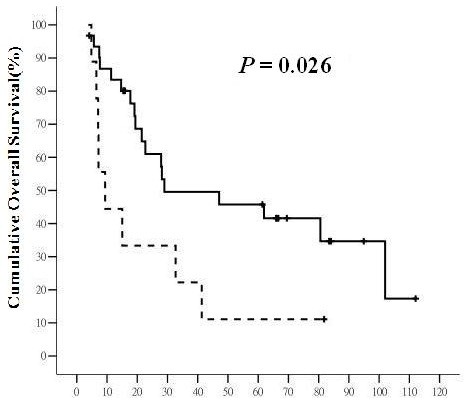
**The overall survival (OS) of hepatocellular carcinoma (HCC) with and without CK19 expression in primary liver tumor.** The solid line represents HCC without CK19 expression in primary liver tumor and the dashed line represents HCC with CK19 expression in primary liver tumor. The horizontal axis is the survival in months and the vertical axis is the percentile cumulative survival. The median OS was 9.37 ± 3.28 (95% CI 2.942 to 15.798) months for tumor with CK19 expression and 28.96 ± 20.96 (95% CI 0 to 70.039) months for tumor without CK19 expression (*P* = 0.026). HCC with CK19 expression in primary liver tumor had a significantly poorer OS.

**Figure 2:**
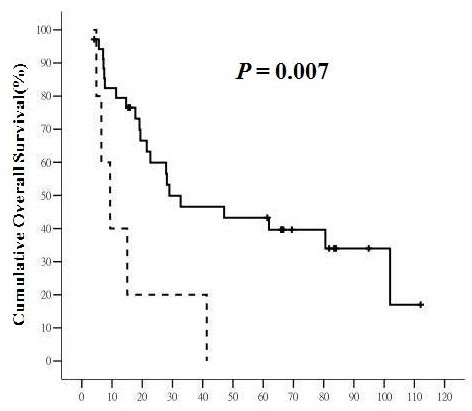
**The overall survival (OS) of hepatocellular carcinoma (HCC) with and without CK19 expression in regional lymph nodes (LN).** The solid line represents LN without CK19 expression and the dashed line represents LN with CK19 expression. The horizontal axis is the survival in months and the vertical axis is the percentile cumulative survival. The median OS was 9.37 ± 3.17 (95% CI 3.165 to 15.575) months for LN with CK19 expression and 28.96 ± 12.92 (95% CI 3.642 to 54.278) months for LN without CK19 expression (*P* = 0.007). HCC with CK19 expression in regional LN had a worst prognosis in terms of OS.

**Figure 3:**
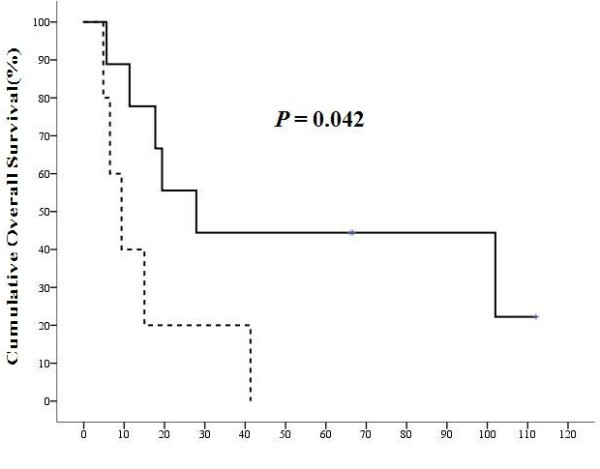
**The overall survival (OS) of N1 hepatocellular carcinoma (HCC) patients with and without CK19 expression in regional lymph nodes (LN).** The solid line represents the OS of CK19 (−) primary liver tumor with CK19(−) lymph node metastasis (LNM) and dashed line represents that of CK19(+) primary liver tumor with CK19(+) LNM. The horizontal axis was the survival in months and the vertical axis was the percentile cumulative survival. The median OS of patients with CK19(+) LNM and CK19(+) primary tumor was significantly poorer than those with CK19(−) LNM and CK19(−) primary tumor (*P* = 0.042). CK19 expression in metastatic LN was a poor prognostic factor for HCC with LNM.

**Figure 4:**
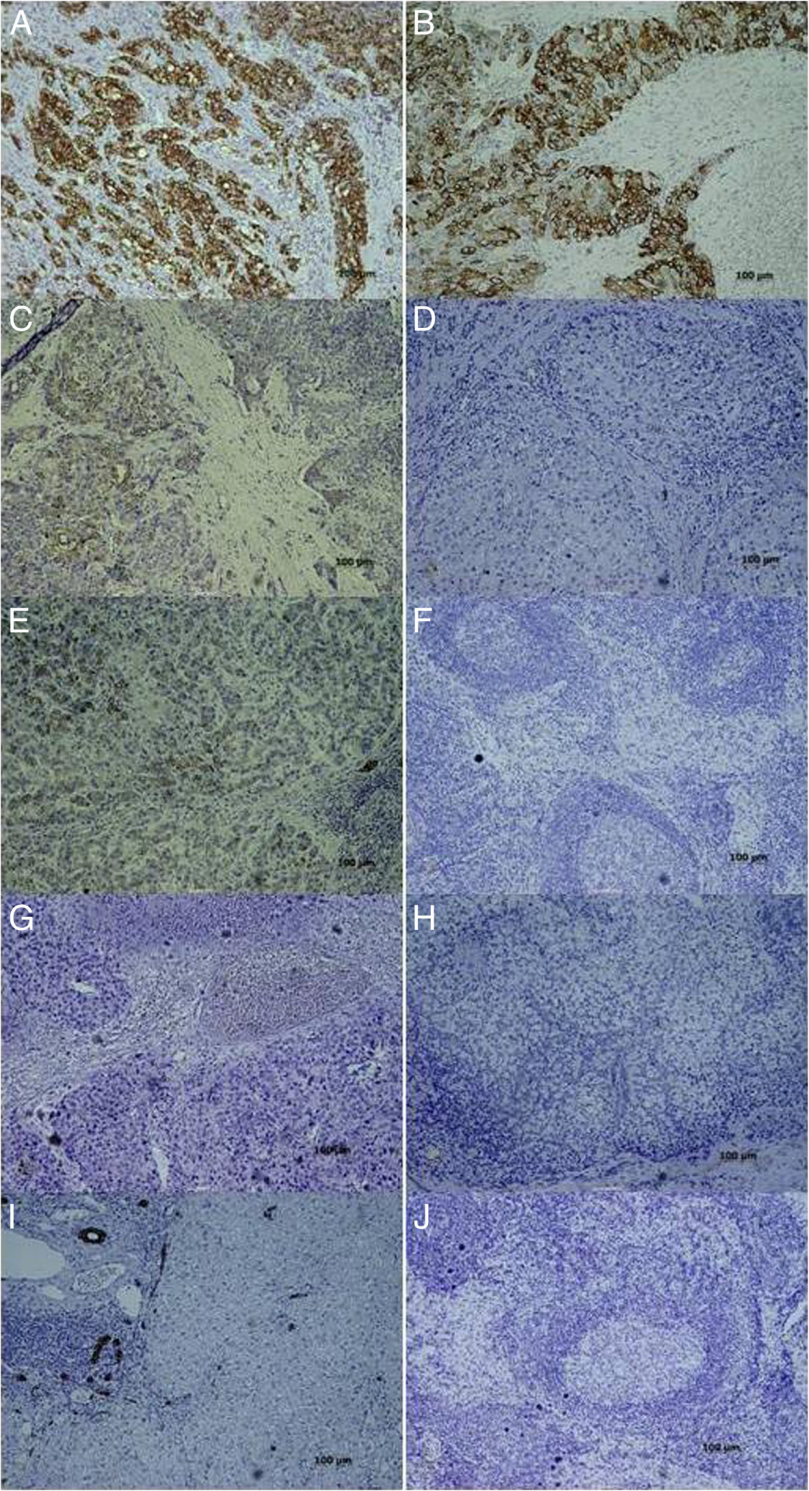
**Immunohistochemical microphotograph of primary liver tumor (left column: A, C, E, G, and I) and regional lymph nodes (LN) (right column: B, D, F, H, and J) for CK19 expression. A** and **B)** CK19 (+) primary liver tumor with CK19 (+) lymph node metastasis (LNM). The cytoplasmic staining of CK19 can be demonstrated in both primary tumor and metastatic LN. **C** and **D)** CK19 (+) primary liver tumor with CK19(−) LNM. The regional lymph nodes had been infiltrated by metastatic tumor cells, which did not express CK19. **E** and **F)** CK19 (+) primary liver tumor without LNM. The normal LN structure can be clearly identified. **G** and **H)** CK19 (−) primary liver tumor with CK19(−) LNM. Both the primary tumor and metastatic LN did not express CK19 in their cytoplasm. On the other hand, the biliary epithelial cells expressed CK19. **I** and **J)** CK19(−) primary liver tumor without LNM. (Magnifications, x100).

Of 42 patients, 26 (61.9%) patients died during the follow-up period, including one patient who died from acute respiratory failure unrelated to HCC or liver cirrhosis. The 5-year OS rate was 32.5%, with median survival time of 22.08 months. Patients with CK19 (+) primary liver tumor or CK 19(+) LN had significantly poorer overall survival than those without CK19 expression (for primary liver tumor, 9.37 ± 3.28 (2.942 to 15.798) months versus 28.96 ± 20.96 (0 to 70.039) months, *P* = 0.026, and for LN, 9.37 ± 3.17 (3.165 to 15.575) months versus 28.96 ± 12.92 (3.642 to 54.278) months, *P* = 0.007, respectively) (Figures 1 and 2). The 5-year OS rates for patients with CK19(+) primary liver tumor or CK19(+) LN were 11.1% and 0%, respectively, while those for CK19(−) primary liver tumor or CK19(−) LN were 45.8% and 43.3%, respectively. Vascular invasion (*P* = 0.005), daughter nodules (*P* = 0.009), positive resection margin (*P* = 0.029), and AJCC tumor stage (*P* = 0.020) significantly decreased overall survival for HCC (Table 2). In patients with N1 disease, although not statistically significant yet, CK19 expression in metastatic LN decreased the OS months (9.37 ± 3.17 versus 27.88 ± 8.25, *P* = 0.068). After classifying these N1 patients into three groups based on the expression of CK19 in primary liver tumor, we found that patients with CK19(+) LNM and CK19(+) primary tumor had a significantly poorer OS than patients with CK19(−) LNM and CK19(−) primary tumor (*P* = 0.042) (Figure 3). Cox regression analysis demonstrated that CK19 expression in regional LN (HR: 5.695 (1.85 to 17.52), *P* = 0.002) and daughter nodules (HR: 2.573 (1.50 to 4.40), *P* = 0.01) were independent poor prognostic factors for overall survival. CK19 expression in primary liver tumor, on the other hand, was not a significant independent prognostic factor after Cox regression analysis (Table 3). Figure 4 is the immunohistochemical microphotograph of primary liver tumor and regional LN.

**Table 2:** Univariate analyses of factors associated with overall survival in hepatocellular carcinoma after hepatectomy

	**Median survival (months)**^**a**^	**5-year OS rate (%)**	***P *value**
**Age (yr)**			
≦**60**	28.96 ± 3.98 (21.165 to 36.755)	40.4	0.338
>**60**	15.02 ± 19.37 (0 to 52.977)	34.6	
**Gender**			
**Male**	22.68 ± 5.44 (12.020 to 33.340)	30.1	0.068
**Female**	28.14 ± 6.45 (15.491 to 40.789)	62.5	
**Alcohol**			
**Yes**	19.40 ± 7.87 (3.968 to 34.832)	18.5	0.027
**No**	47.08 ± 32.31 (0 to 110.413)	46.6	
**Hepatitis B virus**			
**Positive**	28.96 ± 3.74 (21.630 to 36.290)	40.3	0.332
**Negative**	17.72 ± 3.66 (10.556 to 24.884)	32.7	
**Hepatitis C virus**			
**Positive**	47.08 ± 38.99 (0 to 123.488)	41.7	0.665
**Negative**	28.14 ± 5.04 (18.265 to 38.015)	37.2	
**Preoperativeα-fetoprotein (ng/ml)**			
≦**20**	61.91 ± 31.65 (0 to 123.934)	51.3	0.847
>**20**	27.88 ± 6.69 (14.772 to 40.988)	33.4	
**Child-Pugh classification**			
**A**	28.96 ± 7.08 (15.086 to 42.834)	38.7	0.461
**B**	19.40 ± 0 (−− to --)	0	
**Size (cm)**			
≦**5**	61.91 ± 34.99 (0 to 130.486)	58.3	0.310
>**5**	22.68 ± 5.46 (11.988 to 33.372)	30.7	
**Encapsulation**			
**Yes**	47.08 ± 28.79 (0 to 103.515)	48.6	0.122
**No**	19.10 ± 3.34 (12.551 to 25.649)	28.6	
**Tumor rupture**			
**Yes**	27.88 ± 4.99 (18.105 to 37.655)	32.9	0.206
**No**	80.55 ± 46.93 (0 to 172.539)	75.0	
**Vascular invasion**			
**Yes**	17.72 ± 4.26 (9.373 to 26.067)	9.0	0.005
**No**	61.91 ± 31.46 (0.240 to 123.58)	51.3	
**Daughter nodules**			
**Yes**	17.72 ± 8.14 (1.760 to 33.680)	10.8	
**No**	47.08 ± 26.69 (0 to 98.611)	48.6	0.009
**Resection margin**			
**Positive**	7.63 ± 2.17 (3.379 to 11.881)	20.0	0.029
**Negative**	32.71 ± 9.00 (15.067 to 50.353)	40.1	
**Liver cirrhosis**			
**Positive**	28.96 ± 8.66 (11.981 to 45.939)	41.0	0.791
**Negative**	22.68 ± 9.37 (4.322 to 41.038)	33.3	
**Edmonson and Steiner grade**			
**I**	36.91 ± 36.64 (0 to 133.736)	60.0	0.063
**II**	80.55 ± 32.99 (15.895 to 145.205)	60.0	
**III**	22.68 ± 5.46 (11.978 to 33.382)	24.2	
**IV**	17.72 ± 3.20 (11.443 to 23.997)	0	
**Stage**			
**I**	101.98 ± 0 (−− to --)	55.6	0.020
**II**	61.91 ± 22.30 (18.198 to 105.622)	75.0	
**III**	19.40 ± 3.33 (12.873 to 25.927)	22.5	
**IV**	11.34 ± 6.12 (0 to 23.326)	0	
**CK19 liver **^**b**^			
**Positive**	9.37 ± 3.28 (2.942 to 15.798)	11.1	0.026
**Negative**	28.96 ± 20.96 (0 to 70.039)	45.8	
**CK19 LN **^***c***^			
**Positive**	9.37 ± 3.17 (3.165 to 15.575)	0	0.007
**Negative**	28.96 ± 12.92 (3.642 to 54.278)	43.3	

^a^*Median survival±standard error (95% confidence interval).*

^b^*Immunohistochemical staining of primary liver tumor specimen for CK19.*

^c^ Immunohistochemical staining of lymph nodes specimen for CK19.

**Table 3:** Multivariate analyses of factors associated with overall survival in hepatocellular carcinoma after hepatectomy

	** Multivariate analysis**	
	**Hazard ratio (95% CI)**	***P *value**
**Daughter nodules**	2.573 (1.50 to 4.40)	0.001
**CK19 LN**^**a**^	5.695 (1.85 to 17.52)	0.002

^a^Immunohistochemical staining of lymph nodes specimen for CK19.

## Discussion

During development of human liver, hematopoietic stem cells first differentiate into hepatic stem cells (hepatoblasts), then hepatic progenitor cells (HPC), and finally hepatocytes or biliary epithelial cells [19-21]. Cytokeratins, on the other hand, are typical epithelial cell markers that are expressed in a tissue-specific and differentiation-dependent manner [22]. Differences in cytokeratin expression are routinely used to determine the primary cells of origin of malignant tumor [23,24]. In normal human liver, hepatocytes express CK8 and CK18, while biliary epithelial cells express CK7 and CK19 [25]. Hepatic progenitor cells, on the other hand, express markers specific for both hepatocytes and biliary epithelial cells. As progenitor cells differentiate into different cell lineages, they lose specific phenotypic characteristics. For example, CK19 is first lost when hepatic progenitor cells differentiate into hepatocyte lineage [14]. In other words, tumor cells of HCC should not express CK19 if they originate from hepatocytes. However, the current study and several previous reports showed that some HCC can express CK19, the marker specific for biliary epithelial cells [14,15,26-30]. These consistent findings suggested that some HCC may develop from, instead of hepatocytes directly, but hepatic progenitor cells which express CK19 [14,25,31,32]. An earlier study that focused on immunohistologic expression of HCC patients who received hepatectomy indicated that about 10% of HCC with typical microscopic histologic features had cholangiocellular characteristics [14]. The study employed CK19 expression as the cholangiocellular marker and concluded that the cholangiocellular-marker positive HCC may originate from hepatic progenitor cells, or they may acquire the characteristics of cholangiocellular epithelium by metaplasia. Further studies are warranted to investigate the carcinogenesis of this special HCC subtype.

Our study found that CK19 expression in primary liver tumor was associated with a higher incidence of LNM and tumor non-encapsulation. An earlier published work reported that HCC with LNM, though occurring in a very low incidence, tended to be an infiltrating type of HCC and carried an extremely poor prognosis [12]. A study from our group also indicated that HCC encapsulation was associated with less vascular invasion and was a significant prognostic factor for better outcome in larger HCC. These studies suggested that LNM and tumor non-encapsulation were significant indicators for HCC invasiveness and poor outcome. In other words, CK19 expression of primary liver tumor may possibly indicate the worse prognosis for the patient. The survival analysis from the current study further strengthened this implication. Reports done by groups in China and by others stated that increased expression of CK19 in HCC was significantly correlated with LNM and a poorer outcome [14,16,17]. CK19 expression by HCC was also suggested to be a predictor of early postoperative recurrence and was associated with increased invasiveness and lymph node metastasis [16]. Studies focused on molecular classification and diagnosis of HCC also indicated that CK19 expression can be used as a marker of aggressive and advanced HCC [33,34]. Our findings were consistent with these reports and we concluded that: (1) CK19 expression in primary liver tumor was an indicator of HCC invasiveness including LNM and non-encapsulation, and (2) CK 19 expression in primary liver tumor was a poor prognostic factor for overall survival.

Several studies, including the aforementioned ones, demonstrated the impact of CK19 expression by primary tumor on HCC outcome [14-17,25,33,34]. Nevertheless, few investigated the molecular signature of metastatic LN and the influence of these specific molecules on HCC outcome. Our study was the first one in the literature to examine the expression of CK19 by regional LN of HCC. The expression of CK19 in regional LN, which usually did not have epithelial cells, may indicate that the CK19(+) primary liver tumor cell had metastasized from liver to regional LN. We found that seven out of nine (77.8%) CK19 (+) HCC had LNM, and all patients who had positive CK19 expression in their LN (five patients) were also positive for CK19 expression in primary liver tumor. Therefore, we suggested that HCC with immunopositivity of CK19, meaning presence of hepatic progenitor cells, had a high propensity for LNM.

Our study shows that CK19 expression in primary liver tumor or regional LN was associated with poorer overall survival. However, after Cox regression multivariate analysis, only CK19 expression in LN (HR: 5.695 (1.85 to 17.52), *P* = 0.002) and the presence of daughter nodules (HR: 2.573 (1.50 to 4.40), *P* = 0.01) were independent poor prognostic factors for overall survival. In addition, in patients with LNM, CK19 expression in metastatic LN significantly decreased the overall survival, when compared with CK19(−) LNM in CK19(−) primary liver tumor. Subgroup analysis found that there was no significant difference between CK19 (+) LNM and CK19(−) LNM regarding most major clinicopathological factors. This indicated that the worst prognosis of CK19 (+) LNM was not attributed to other prognostic factors, but only to CK19 expression in metastatic LN. Our study, therefore, was the first report in the English literature to demonstrate the worst prognostic impact of CK19 (+) LNM on HCC after hepatectomy. The nature of CK19 (+) primary liver tumor with CK19(−) metastatic LN remains uncertain and mandates further investigation. As shown above, HCC with LNM carried a poor outcome [12]. Our study further explored the possibility that HCC with CK19(+) metastatic LN may be an HCC subtype with even worse prognosis. However, with appropriate treatment and follow-up, this subset of patients still can achieve a median overall survival of 9.37 ± 3.17 months after hepatectomy. Our earlier study demonstrated that LNM may not be a contraindication for curative resection for HCC. A more aggressive surgical treatment including lymph node dissection was suggested when LNM was suspected [12]. The present study echoed that finding and we further suggested that CK19 immunohistochemical staining of primary liver tumor and dissected LN should be performed after hepatectomy to determine LN status and to predict outcome.

This study had some drawbacks. First, the rarity of HCC with LNM resulted in a small sample size and rendered statistical analysis difficult. We believe that with a larger sample size, our findings will become even more significant and persuasive. Second, since this study was a retrospective hospital-based analysis, incomplete data collection were inevitable when reviewing records from a very long time ago. Selection bias might also exist when enrolling patients into this study. In addition, there is fundamental problem in using immunohistochemical staining in evaluating the expression profile of CK19. It requires experienced histopathologists for staining and interpreting the immunohistochemical examination result. Other techniques, including polymerase chain reaction, could be more objective and sensitive. Therefore, a well-designed prospective study with long-term follow-up is required to further validate our study.

## Conclusions

In conclusion, our study showed that (1) CK19 expression in primary liver tumor is an indicator of HCC invasiveness including LNM and non-encapsulation, (2) CK19 expression in metastatic LN of primary HCC iss associated with an extremely poor outcome after operation, and (3) CK19 immunohistochemical staining of primary liver tumor and regional LN should be performed after hepatic resection to determine LN status and to predict outcome. Since CK19 was considered a marker of hepatic progenitor cells and biliary epithelial cells, the subset of HCC expressing CK19 in metastatic LN may represent an HCC category that originates from HPC or by metaplasia and carries an extremely poor prognosis. It is of clinical significance to identify these patients at risk for more aggressive surgical treatment and postoperative follow-up. A recent study also indicated that the activation of the epidermal growth factor (EGF)-EGF receptor signaling pathway is associated with the development of CK19 (+) HCC, and this pathway may account for the poor prognosis of patients [35]. The mechanism by which EGFR signaling pathway induces CK19 expression remains unknown. Further studies are warranted to elucidate the carcinogenesis of this subset of HCC.

## Consent

Written informed consent was obtained from the patient for publication of this report and any accompanying images.

## Abbreviations

AJCC: American Joint Committee on Cancer; HBV: Hepatitis B virus; HCC: Hepatocellular carcinoma; HCV: Hepatitis C virus; HPC: Hepatic progenitor cells; ICC: Intrahepatic cholangiocarcinoma; LN: Lymph node; LNM: Lymph node metastasis; OS: Overall survival.

## Competing interests

The authors declare that they have no competing interests.

## Authors’ contributions

CWL analyzed the clinico-pathological data and drafted the manuscript. WLK collected the clinical data and revised the manuscript. MCY designed and coordinated the study. TCC carried out IHC examination and interpreted the results. CNT designed the study and analyzed the clinical data. WCL revised the manuscript. MFC coordinated the study and revised the manuscript. All authors read and approved the final manuscript.
